# Orientation-Independent T2 Mapping Enhances MRI-Based Cartilage Characterization

**DOI:** 10.1007/s10439-025-03774-3

**Published:** 2025-06-17

**Authors:** Henri P. P. Leskinen, Juuso Tuppurainen, Jiri Jäntti, Janne T. A. Mäkelä, Ervin Nippolainen, Isaac O. Afara, Juha Töyräs, Olli Nykänen, Mikko J. Nissi

**Affiliations:** 1 Department of Technical Physics, University of Eastern Finland, Yliopistonranta 8, 70210 Kuopio, Finland; 2 Mikkeli Central Hospital, Mikkeli, Finland; 3 Diagnostic Imaging Center, Kuopio University Hospital, Kuopio, Finland; 4 School of Electrical Engineering and Computer Science, The University of Queensland, Brisbane, Australia; 5 Science Service Center, Kuopio University Hospital, Kuopio, Finland

**Keywords:** Quantitative MRI, Osteoarthritis, T2 relaxation, T2 anisotropy, Orientation-independent T2

## Abstract

**Purpose:**

Quantitative *T2* mapping is an important MRI method for assessing degenerative changes in articular cartilage. Recently, in a measurement setup with automated sample re-orientation, it was demonstrated that T2 can be split into its orientation-independent components. This quantitative MRI study aims to assess the diagnostic significance of the automated approach with *ex vivo* human cartilage.

**Methods:**

*T2* maps of 30 human osteochondral samples harvested from 5 cadaveric individuals were acquired at 9.4T in 13 orientations, allowing calculation of the *T2* components. Additionally, *T1*, adiabatic *T1ρ*, and continuous wave *T1ρ* with two spin-lock frequencies were scanned in a single orientation. For reference, the collagen network anisotropy, proteoglycan content and biomechanical indentation properties were measured. The relationships between quantitative MRI and reference parameters were studied using Mann-Whitney U-test and Spearman’s rank correlation. All parameters were compared between healthy and degenerated groups based on OARSI grading.

**Results:**

The anisotropic relaxation rate component of *T2* (*R2a*), and all *T1* and *T1ρ* parameters differed (p < 0.05) between the groups in superficial cartilage. *R2a* correlated moderately with PLM anisotropy (r = 0.44) and optical density (r = − 0.37) in the deep zone. Isotropic *T2* component (*R2i*) correlated with instantaneous modulus (r = 0.48), and *R2a* with phase shift between stress and strain during indentation testing (r = − 0.44). *T1* and *T1ρ* parameters correlated with both, instantaneous and dynamic modulus in several zones of cartilage.

**Conclusion:**

The elevation of *T2* in degenerated cartilage is primarily driven by the *R2a* component, whereas the *R2i* component showed no significant difference between healthy and degenerated human articular cartilage.

## Introduction

### Articular Cartilage and Osteoarthritis

Articular cartilage enables frictionless movement of joints and protects them by distributing stresses and strains [1]. Articular cartilage consists mainly of water, collagen fiber network, proteoglycans (PG), and chondrocytes. Adult articular cartilage can be divided into three distinct layers based on its structure and composition. In the superficial zone (SZ), collagen fibers are tightly packed and aligned parallel to the surface. Below the SZ is a transitional zone (TZ), and occasional additional TZ-layers [2]. In the deep zone (DZ), which is the thickest of the layers, collagen fibrils are oriented perpendicular to the bone-cartilage-interface and are highly organized.

Osteoarthritis (OA) is a common disease affecting approximately 7.6% of the global population [3]. The global economic costs of OA exceed US$100 billion and it has substantial effect on quality of life on individual level [3]. OA can be idiopathic or post-traumatic and is more common among the elderly population. In clinical practice, most prevalent are hip, knee, hand, foot and spine osteoarthritis [4]. Current diagnosis methods rely on observing signs of advanced disease in radiographic images such as narrowed joint space due to cartilage loss, osteophytes and subchondral sclerosis. Earlier diagnosis would allow conservative intervention earlier which in turn would result in an increase of healthy years lived [5].

In the early stages of OA, articular cartilage undergoes microscopic structural [6] and compositional [7] changes. Loss in proteoglycan (PG) content and decreased integrity of the collagenous network are among the most dominant of these changes [4, 8]. Proteoglycans and the collagenous network provide structural integrity for cartilage, which is why cartilage mechanical properties are also impaired in early stage OA [9]. One of the earliest detectable changes in OA is the initial fibrillation of superficial collagen network. Method for detecting early-stage OA should be sensitive to at least one of these changes.

### qMRI of Articular Cartilage

Elevation in *T2* has been linked to a loss of collagen structure integrity, which in turn, together with loss of PGs is typical in early OA [8, 10, 11].

The highly organized collagenous network gives structure to articular cartilage and makes it orientation-dependent (i.e. anisotropic) in certain imaging modalities, such as polarized light microscopy (PLM) and MR imaging [12–14]. The anisotropy in MRI arises from the collagen fibers restricting water diffusion in highly organized tissues such as cartilage. This causes changes in residual dipolar coupling resulting in an orientation-dependent change of *T2* relaxation time [15, 16]. Due to the phenomenon, just the orientation of the imaged cartilage (or other highly organized tissue) causes substantial variation also in non-quantitative MRI signal in everyday clinical practice.

In the last three decades, several models for the orientation dependence of *T2* with respect to the main magnetic field (B0) have been suggested [17, 18]. Orientation-independent anisotropic *R2a* and isotropic *R2i* components (*R2 = 1/T2*) together with total orientation-independent *T2* (*T2tot*) and *T2* relaxation anisotropy can be estimated from a multi-orientation scan as described previously [19].

### Ex vivo Reference Methods

Structural, compositional and mechanical properties of articular cartilage can be determined experimentally most accurately *ex vivo*. PLM allows defining of collagen fiber network anisotropy, birefringence, and fiber angle [12]. PLM has previously been used for also qualitative assessment of degenerative status of cartilage and demonstrated its effectiveness for identifying collagen alterations not evident in the traditional scoring systems [20]. Digital densitometry (DD) microscopy allows defining the optical density of a Safranin-O stained histological sections, which in turn, is a surrogate marker for cartilage PG content [21]. The functional properties of cartilage can be further assessed via biomechanical testing by measuring, e.g., strain-dependent instantaneous modulus (*E*_ε_) and dynamic modulus (*E*_dyn_). Changes in these parameters have been linked to OA [22–24].

### Aim and Hypothesis

We hypothesize that orientation-independent *T2* parameters (*R2i*, *R2a* and *T2* anisotropy) are sensitive to structural and compositional changes in collagen network integrity and anisotropy, and possibly in PG content, observed in early OA. Since *T2* is shown to be sensitive to changes in cartilage collagen matrix, *R2a* and *T2* anisotropy might be of high importance in MRI-based characterization of early-stage OA. PLM, DD, and biomechanical indentation testing are used as reference methods due to their established sensitivity to the collagen matrix anisotropy, PG content, and biomechanical moduli, respectively, properties of biomechanical significance in articular cartilage. Finding correlation between qMRI parameters and these reference methods could provide novel insight into the role of the studied orientation-independent qMRI parameters in OA.

## Materials and Methods

### Samples

The samples used in this study were osteochondral plugs (*d* = 4mm, *n* = 30) prepared from cadaveric human distal femur of five individuals (47–71 years, BMI 27 ± 4) and stored at − 22 °C prior to experiments. The sample plugs were harvested from both load-bearing, and non-load-bearing locations. The joints were obtained from a commercial biobank (Science Care, USA) and the study has received ethical permission (North Savo Hospital district (PSSHP) Ethical board permission 134/2015). Previous publications on this sample set have focused on classification and analysis of infrared spectroscopy spectra [25]. Eight of the 16 healthy samples were from the youngest individual (47 years). Four of the five individuals had at least one OARSI grade 0 sample. The youngest individual had also two OARSI 1 graded samples.

### MRI

**Fig. 1:**
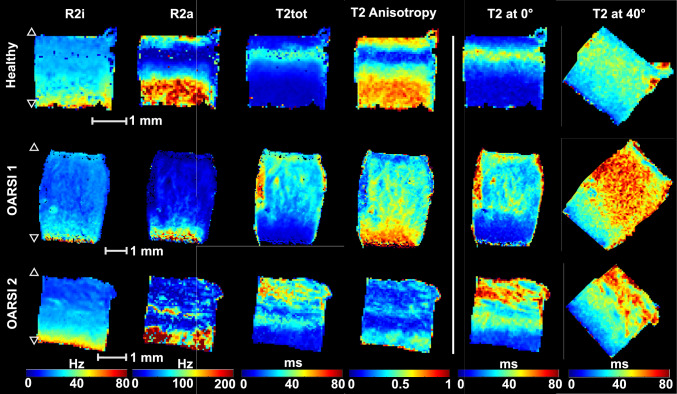
*T2* parameter maps for healthy and degenerated samples. *R2a*, *T2tot*, *T2* at 0° and *T2* Anisotropy showed difference between SZ of healthy and degenerated samples. Changes in the appearance of the zonal structure are among the most prominent changes. **△** = articular surface. ▽ = bone-cartilage interface. Please note the varying scale between samples.

Multi-orientation MRI scans were conducted for the osteochondral plugs to enable calculating orientation-independent *T2* parameters (Fig. 1). The scanning was conducted at 9.4T using VnmrJ 3.1 Varian/Agilent DirectDrive console (Varian Associates Inc., Palo Alto, CA, USA) and a 19-mm quadrature RF volume transceiver (RAPID Biomedical GmbH, Rimpar, Germany). The samples were thawed at room temperature and immersed in perfluoropolyether (Galden HS 240, Solvay Solexis, Italy) to provide a signal-free background. The samples were placed in a 3-D printed holder coupled to a motorized microcontroller-driven (Arduino Micro A000053) system that allowed reorienting the sample automatically between the scans. The samples were scanned at 13 orientations along a single plane, spanning 180° with respect to B0. Sample orientation refers to the angle between the B0 field and cartilage surface normal. Samples were scanned with T2-MESE (Multi-Echo Spin-Echo) sequence with a matrix size of 256 × 256 and field of view of 13 × 13mm (pixel size = 50.8 µm, *N*_echos_ = 10, echo spacing = 5.516ms). Monoexponential *T2* maps were calculated for each orientation. All orientations were co-registered to the first orientation of each sample using Elastix [26]. A model of anisotropic *T2* relaxation [19] was fit to the data and voxel-wise *T2* anisotropy maps of each sample were calculated using Michelson contrast *A* = *(R2*^max^*–R2*^min^*)/ (R2*^max^ *+ R2*^min^*)* [19, 27]. Regions of interest (ROIs) for the SZ, TZ, and DZ were defined based on identifying TZ in the *T2* anisotropy map, utilizing asymmetric bell-shape curve method [2], and defining cartilage above it as SZ, and below it as DZ [28, 29]. Bulk average over all pixels was also calculated. Four additional sequences were scanned in the first orientation. These sequences were: inversion recovery IR-*T1* (repetition time TR = 7 s, inversion time TI = 0.2, 0.5, 0.8, 1.1, 1.4 and 3 s), *T1ρ* measured using adiabatic pulses (*Ad-T1ρ*) (TR = 5 s, pulse shape = HS1, R = 10, τ_p_ = 4.5ms, and γB_1,max_/2π = 2.5 kHz, pulse trains of 0, 4, 8, 12, 24 and 36 pulses using MLEV4 phase cycling), and two continuous wave (CW-)*T1ρ* [30] sequences (TR = 5 s, γB_1_/2π = 500 and 1000 Hz, spin-lock durations of 0, 8, 16, 32, 64 and 128 ms).

### Biomechanical Indentation Test

Strain-dependent instantaneous modulus (*E*_*ε*_) was determined using a previously presented stress-relaxation protocol and 1 Hz dynamic modulus (*E*_dyn_) and stress-strain phase shift (*θ*) were determined using dynamic protocols [23]. The measuring setup consisted of a displacement actuator with a resolution of 0.1 µm (PM1A11939, Newport, Irvine, CA, USA) and a load cell of 0.25 kg (Model 31, Honeywell International Inc., Charlotte, NC, USA). A goniometer was applied to ensure perpendicularity between cartilage surface and plane-ended cylindrical indenter. The diameter of the indenter was 0.55 mm. The stress-relaxation testing protocol included four 5% strain steps, each of them followed by 900 s relaxation time. Strain rate was set to 100%/s relative to cartilage thickness. A cyclic loading protocol was applied using a 1 Hz sinusoidal waveform for 4 cycles, with a peak-to-peak strain amplitude of 4% of the remaining cartilage thickness [23].

### Histological Analysis (OARSI, PLM & DD)

After the experiments, the samples were fixed in 10% formalin, decalcified in EDTA and dehydrated in graded alcohols, embedded in paraffin and processed into histological slices. Unstained microtomy slices of 5 μm in thickness were produced for PLM, and Safranin-O-stained slices for DD and light microscopy. Safranin-O staining was performed by immersing all the slices in the staining solution in the same batch. Possible over- and under-staining due to slight variation in slice thickness was compensated by using an average of three slices in DD measurements. Studies have shown that the PG content, estimated via OD from slices prepared using the method utilized in this study, correlates with histochemical glycosaminoglycan content [31], which, in turn has been linked to cartilage biomechanical properties [23, 32]

Fiber anisotropy was defined with PLM as the Michelson contrast [12]. Measurements were conducted using an *ePLM* set up where the sample is mounted on a path of monochromatic light source between two crossed polarizers that can be turned synchronously [12, 19]. A 0.344 mm wide column ROI was selected from the PLM anisotropy map and used for calculating an average depth-wise profile. Profile was segmented visually into three zones similarly to the MRI analysis using PLM anisotropy maps. Earlier studies show that this analysis provides a reasonable agreement between the MRI and histological ROI selection [2]. In addition, segmentation results were compared with MRI to ensure reliable outcome.

DD was conducted to measure optical density of the Safranin-O stained sample slices using a charge-coupled device camera, and a monochromator [33]. Obtained grayscale images were converted to optical density images using calibration filters. After DD, light microscopy was used to define the degeneration level for each sample. The degeneration level was defined as an OARSI score using pseudonymized sample names according to Pritzker et al. 2006 [34] as a consensus of three independent evaluators.

### Statistical Analysis

The samples were divided into two groups based on their OARSI grading. The healthy group consisted of 16 samples of OARSI grade 0. The degenerated group consisted of 14 samples with the OARSI grades of 1 (*n* = 10), 2 (*n* = 3) and 3 (*n* = 1). OARSI grade 0 is healthy and grades 1 through 3 can be considered mild or early OA [34].

qMRI data was not normally distributed based on Shapiro-Wilk normality test. Thus, Mann-Whitney U-test was performed to study differences between the healthy and degenerated groups using IBM SPSS 27. Spearman correlation was calculated between qMRI parameters (*R2i*, *R2a*, *T2tot*, *Ad-T1ρ*, *CW-T1ρ* SL (spin lock) = 500Hz. *CW-T1ρ* SL = 1000Hz and *T1*) and reference methods (PLM anisotropy, optical density, biomechanical moduli).

## Results

The average thicknesses of SZ, TZ and DZ, determined using PLM, were 0.31 ± 0.19 mm, 0.47 ± 0.22 mm and 1.39 ± 0.58 mm respectively, corresponding to roughly 6, 9 and 28 pixels in MRI.

**Fig. 2:**
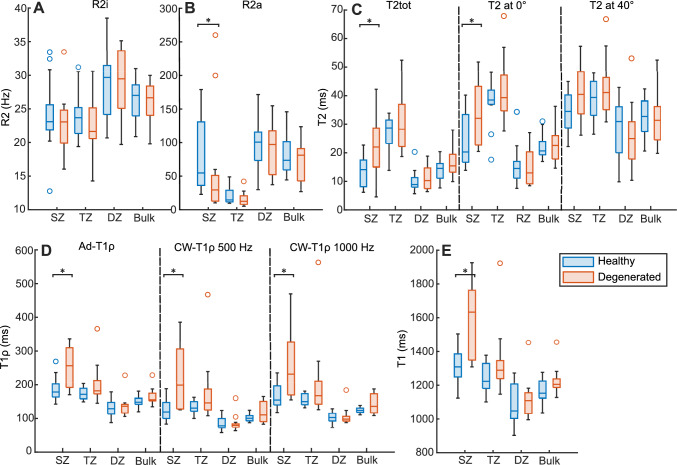
Boxplot of qMRI parameters in the superficial zone and bulk cartilage in the two sample groups. **A** Isotropic and **B** anisotropic R2, **C** T2, **D** Adiabatic and Continuous wave T1ρ, and E) T1. Statistically significant findings indicated with an asterisk (*).

The orientation independent *T2* parameter maps (*T2tot*, *R2a*, *R2i*, and *T2* anisotropy) differed visually between the healthy and degenerated samples (Fig 1.). The anisotropic *T2* relaxation rate component (*R2a*) was observed to be significantly lower in the SZ of degenerated samples. *R2i* did not differ between the groups (Table 1). Additionally, all four *T1* and *T1ρ* parameters demonstrated statistically significant increase in the SZ of degenerated cartilage (Fig. 2). Single-orientation *T2* scans at 0° and ~ 40° were chosen to represent situations with different magic angle effects (Table 1). Difference between the SZ of healthy and degenerated cartilage was observed in a single orientation *T2* at 0°, but not at 40° orientation. Orientation-independent *T2tot* showed statistically significant elevation in the SZ of degenerated cartilage.

**Table 1:** p-values (Mann-Whitney U-test) for the statistical comparison between healthy and degenerated cartilage for qMRI parameters, PLM anisotropy, optical density, and biomechanical properties.

	SZ	TZ	DZ	Bulk
*R2a*	0.022*	0.179	0.790	0.498
*R2i*	0.822	0.473	0.637	0.951
*T2* tot	0.013*	0.448	0.580	0.179
*T2* at 0°	0.008*	0.759	0.822	1
*T2* at 40°	0.064	0.355	0.355	0.637
*T2* anisotropy	0.077	0.951	0.790	0.984
*Ad*-*T1ρ*	0.002*	0.313	0.984	0.142
*CW-T1ρ* 500 Hz	0.003*	0.240	0.918	0.224
*CW-T1ρ* 1000 Hz	0.004*	0.400	0.525	0.240
*T1*	< 0.001*	0.257	0.728	0.101
PLM anisotropy	0.886	0.697	0.951	
Optical density	0.854	0.728	0.0582	0.043*
*θ* 1 Hz				0.019*
*E*_dyn_ 1 Hz				0.608
*E*_ε_				0.918

Statistically significant findings (p < 0.05, null hypothesis: the compared groups are from the same sample pool) are indicated with an asterisk (*).

*SZ* Superficial zone, *TZ* Transitional zone, *DZ* Deep zone, *CW* Continuous wave, *Ad* Adiabatic, *I* Isotropic, *a* Anisotropic, *θ* Phase shift in dynamic testing protocol, *Edyn* Dynamic modulus, *Eε* Instantaneous modulus.

**Fig. 3:**
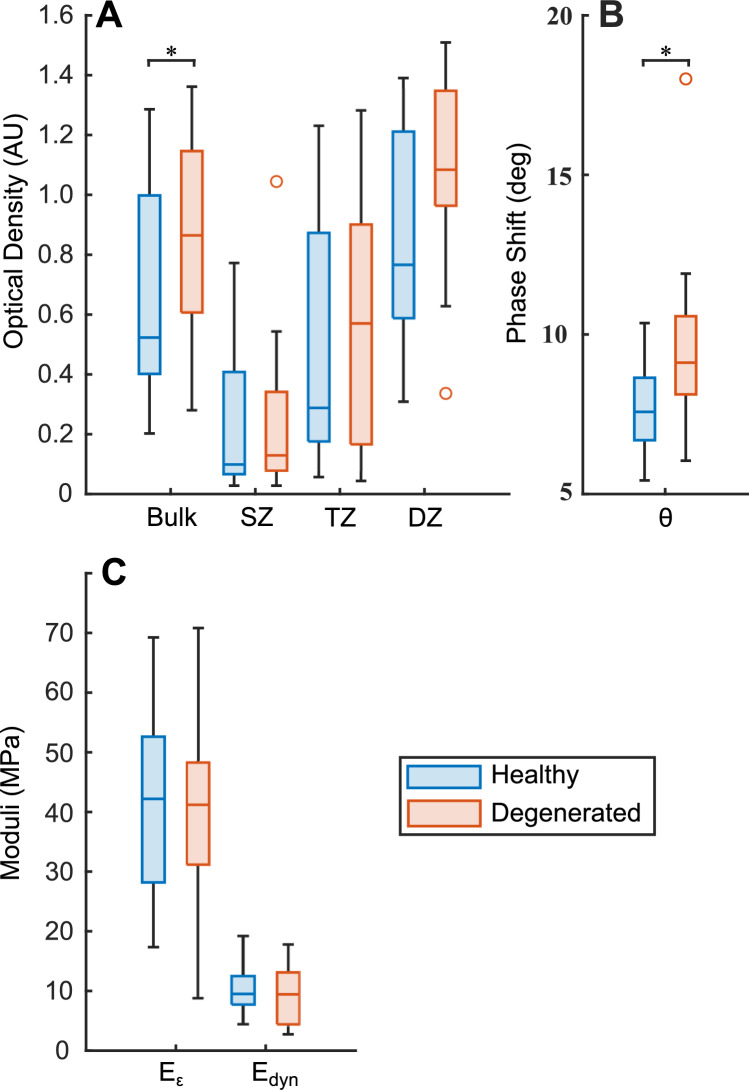
**A** Optical density **B**, phase shift, and **C** moduli results in healthy and degenerated cartilage presented as boxplots in the healthy and degenerated groups. Phase shift at 1 Hz dynamic mechanical testing showed statistically significant difference between healthy and degenerated cartilage. Statistically significant findings are indicated with an asterisk (*).

Neither the strain-dependent instantaneous modulus nor the dynamic modulus demonstrated capability of separating the two groups. However, in the phase shift at 1Hz dynamic mechanical testing there was a statistically significant difference between the healthy and degenerated cartilage (Table 1, Fig. 3). Quantitative PLM anisotropy, without additional qualitative visual inspection appeared insufficient for determining the degenerative status of cartilage. DD measurements showed statistically significant elevation of OD in the degenerated sample group Table 1, Fig. 3).

**Fig. 4:**
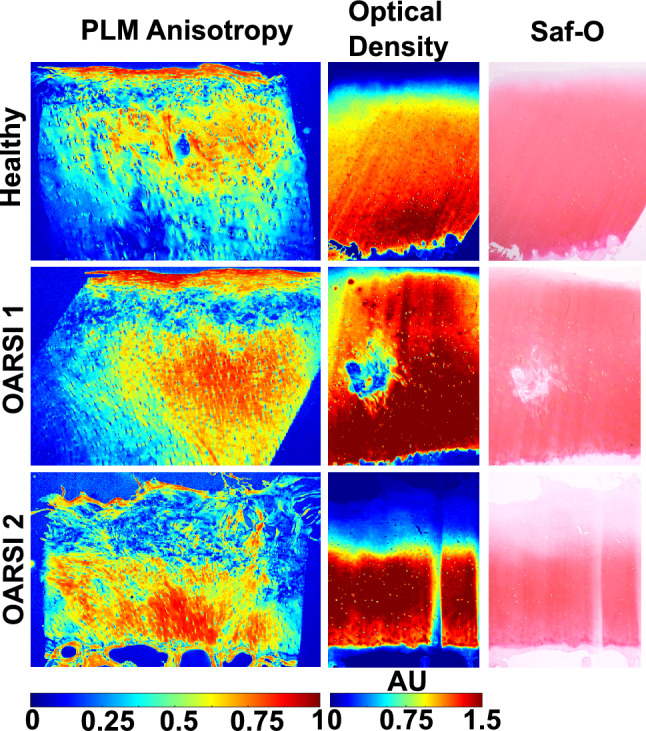
PLM anisotropy, optical density, and light microscopy of Saf-O -stained slices in samples with three different degeneration levels. All histological slices were parallel to each other and within 30 µm.

*T2* anisotropy did not show correlation with PLM anisotropy (Table 2). *T2tot* showed moderate but significant (*ρ* = − 0.47) correlation with PLM anisotropy in the DZ (Table 2, Fig 4). No correlation was found between the *E*_dyn_ and *T2*-based qMRI parameters (Table 3). Instead, a moderate correlation was observed between *R2i* and *E*_ε_. Additionally, DZ and bulk *R2a* and *T2tot* values were found to moderately correlate with the phase shift at 1Hz dynamic mechanical testing. Several moderate correlations were also found between the *T1* and *T1ρ* parameters and *E*_*ε*_ and *E*_dyn_ (Table 3).

**Table 2:** Spearman correlation between qMRI parameters and optical density and PLM anisotropy.

	SZ	TZ	DZ	Bulk
	Optical Density			
*T2* anisotropy	− 0.28	− 0.23	− 0.32	− 0.03
*R2i*	− 0.01	− 0.27	− 0.32	− 0.63*
*R2a*	− 0.40*	− 0.25	− 0.37*	− 0.12
*T2tot*	0.42*	0.28	0.37*	0.12
*T2* at 0°	0.28	0.31	− 0.01	0.21
*T2* at 40°	− 0.08	0.39*	0.41*	0.44*
*Ad*-*T1ρ*	0.12	0.28	0.38*	0.56*
*CW-T1ρ* 500 Hz	0.18	0.36*	0.12	0.01
*CW-T1ρ* 1000 Hz	0.17	0.34	0.13	0.17
*T1*	0.13	0.20	0.45*	0.68*
	PLM anisotropy			
*T2* anisotropy	0.10	− 0.02	0.15	
*R2i*	0.31	0.16	− 0.04	
*R2a*	0.12	0.05	0.44*	
*T2tot*	− 0.13	− 0.06	− 0.47*	
*T2* at 0°	− 0.47	− 0.23	− 0.26	
*T2* at 40°	− 0.05	− 0.05	0.28	
*Ad*-*T1ρ*	− 0.26	− 0.21	− 0.07	
*CW-T1ρ* 500 Hz	− 0.47*	− 0.25	− 0.23	
*CW-T1ρ* 1000 Hz	− 0.37*	− 0.21	− 0.15	
*T1*	− 0.31	− 0.25	− 0.02	

Statistically significant findings (p < 0.05) are indicated with an asterisk (*).

*SZ* Superficial zone, *TZ* Transitional zone, *DZ* Deep zone, *CW* Continuous wave, *Ad* Adiabatic, *I* Isotropic, *a* Anisotropic

**Table 3:** Spearman correlation between qMRI parameters and biomechanical properties.

	SZ	TZ	DZ	Bulk
	*E*_ε_			
*R2i*	0.20	0.44*	0.44*	0.48*
*R2a*	0.25	0.07	− 0.33	− 0.30
*T2tot*	− 0.29	− 0.22	0.25	0.24
*T2* at 0°	− 0.39	− 0.37	0.33	0.32*
*T2* at 40°	0.27	− 0.33	− 0.05	− 0.03
*Ad-T1ρ*	− 0.06	− 0.40*	− 0.44*	− 0.21
*CW-T1ρ* 500 Hz	− 0.36*	− 0.52*	0.07	0.12
*CW-T1ρ* 1000 Hz	− 0.20	− 0.46*	− 0.16	0.05
*T1*	− 0.17	− 0.39*	− 0.57*	− 0.40*
	*E*_dyn_ 1 Hz			
*R2i*	0.15	0.13	0.25	0.28
*R2a*	0.19	− 0.13	0.13	0.10
*T2tot*	− 0.19	0.07	− 0.16	− 0.03
*T2* at 0°	− 0.48	− 0.20	− 0.37	− 0.31
*T2* at 40°	0.01	− 0.05	− 0.19	− 0.23
*Ad-T1ρ*	− 0.38*	− 0.50*	− 0.38*	− 0.61*
*CW-T1ρ* 500 Hz	− 0.44*	− 0.38*	− 0.32	− 0.35
*CW-T1ρ* 1000 Hz	− 0.41*	− 0.54*	− 0.37*	− 0.44*
*T1*	− 0.39*	− 0.60*	− 0.31	− 0.48*
	*θ* 1 Hz			
*R2i*	0.09	0.13	0.06	0.04
*R2a*	− 0.11	− 0.06	− 0.46*	− 0.44*
*T2tot*	0.14	− 0.07	0.44*	0.37*
*T2* at 0°	0.21*	− 0.12	0.30*	0.33
*T2* at 40°	0.15	0.06	− 0.07*	− 0.01*
*Ad-T1ρ*	0.36	0.08	0.07	0.33
*CW-T1ρ* 500 Hz	0.19	− 0.20	− 0.02	0.11
*CW-T1ρ* 1000 Hz	0.27	− 0.06	− 0.04	0.22
*T1*	0.36*	0.10	0.02	0.22

Statistically significant findings (p < 0.05) are indicated with an asterisk (*).

*SZ* Superficial zone, *TZ* Transitional zone, *DZ* Deep zone, *CW* Continuous wave, *Ad* Adiabatic, *I* Isotropic, *a* Anisotropic, *θ* Phase shift in dynamic testing protocol, *Edyn* Dynamic modulus, *Eε* Instantaneous modulus.

## Discussion

This study focused on assessing *T2* relaxation time and its anisotropic, *R2a*, and isotropic, *R2i*, components in healthy and degenerated human articular cartilage *ex vivo*. *T2* relaxation time was elevated in degenerated cartilage as reported previously [8]. Relaxation time increase in degenerated cartilage was observed for the *R2a* component, suggesting that the elevation of *T2* relaxation time is mainly due to changes in its anisotropic component. Since *T2* has been shown sensitive to the collagenous network restricting dipolar interaction [8], and insensitive to changes in PG content, it is reasonable to assume that the *R2a* component reflects the properties of the collagen matrix.

Elevation of *T2* in degenerated cartilage was observed in the 0-degree orientation, but not in the 40-degree orientation, demonstrating the importance of prior information about collagen fiber structure when *T2* mapping is used for OA assessment.

The phase shift between stress and strain in dynamic mechanical testing has been reported to increase as PG content decreases and to decrease as collagen content decreases [23]. This occurs because PGs bind water and enhance fluid-based energy dissipation, while collagen increases the solid (elastic) response of cartilage. Here, phase shift was higher in the degenerated cartilage, suggesting PG loss in the degenerated samples. However, DD findings suggested higher PG content in the degenerated samples, suggesting a more complex pattern of degeneration.

Dynamic modulus has been reported to decrease in severe OA [35]. However, in this study, no significant differences between healthy and degenerated groups were observed in the instantaneous or dynamic moduli. This result might have been different if the sample set included more severely degenerated samples. Several qMRI-parameters studied here seemed to have potential for detecting degenerative changes in cartilage. Given the sensitivity of *T2* to collagen fibers, we hypothesized that the *T2*-components would correlate either with the dynamic modulus, phase shift or both, but instead only moderate correlations were found with the strain-dependent instantaneous modulus (Table 3).

### qMRI

*T1* and *T1ρ* -parameters demonstrated more substantial changes in degenerated cartilage when compared to *R2a* or *T2tot*. Estimation of *T2* components requires several times more data and additional calculations, which magnifies the noise. This might result in masking of small changes under the noise. In previous studies, *T1*, *Ad-T1ρ* and *CW-T1ρ* have been shown to be sensitive to, *e.g.*, post-traumatic OA changes in animal studies [36]. Elevated *T1ρ* has been linked to the degenerative status of human cartilage under load *ex vivo* [37]. However, *T2*, *T1*, *Ad-T1ρ* and *CW-T1ρ* have demonstrated potential for differentiation of early and advanced OA groups [35]. Elevated *T2* and T1ρ have also been linked to OA *in vivo* in humans [38]. Hence, it is reasonable to assume these results to be replicated in spontaneously degenerated human cartilage. Changes in these parameters have been observed predominantly in the SZ [36, 39] and the measurements done in the present study at 0-degree (*T2*, *T1*, *Ad-T1ρ* and *CW-T1ρ*) align with the previous findings. *R2a* likely reflects the properties of the collagen matrix responsible for the relaxation anisotropy. Changes in the collagen matrix seem to remain undetected in early OA via multi-orientation *T2* mapping. *R2i*, on the other hand, seems to be largely insensitive to the collagen matrix integrity, but shows significant association to OD measured using DD.

Several qMRI parameters demonstrated a clear difference between healthy and degenerated sample groups in the SZ, unlike DD or PLM. Since the degenerated sample group predominantly consists of samples having OARSI grade of 1, these findings suggest that qMRI can effectively detect early signs of cartilage degeneration in the SZ.

*T1* and *R2i* showed similarities: *T1* correlated positively and *R2i* negatively with DD and *E*_ε_. This is expected, as both parameters are isotropic [27]. Reciprocity of the relaxation rate and time explains the opposite signs in the correlations. However, *R2i* did not separate degenerated and healthy cartilage. The finding suggests that *T1* is more sensitive to the changes in early OA. *E*_ε_ correlated with qMRI parameters (*R2i*, *T1*, *Ad-T1ρ* and *CW-T1ρ*) mainly in the TZ and DZ, while *E*_dyn_ showed correlation also in the SZ. The lack of correlation between qMRI parameters and *E*_ε_ in the SZ could be explained if *E*_ε_ is not sensitive to certain changes in the surface of the cartilage that the qMRI parameters can detect. In addition, *R2a* and *T2tot* both correlated with the phase shift and PLM anisotropy. Similarities in these parameters are expected since *T2tot* is defined using *R2a*. Since *T2tot* comprises of *R2i* and *R2a*, where *R2a* is mainly due to the anisotropy of the collagenous network. *R2i* can thus be considered as a baseline component of *R2*, reflecting contributions from factors other than collagen anisotropy, such as unbound water, cells, proteoglycans and other tissue microarchitectural factors. Safranin-O density is associated with PG (and GAG) content, which in turn is associated with cartilage hydration level or water content. Hence, cartilage hydration level, and furthermore PG content, are possibly contributors in *R2i*.

Precise definition of *T2* anisotropy requires knowledge of maximum and minimum relaxation times. These are obtained at the magic angle and 0 degrees fiber-to-field angles, respectively. If the scanned orientations do not cover the full dynamic range of *T2*, the anisotropy of *T2* may be underestimated. To avoid this issue, the *T2* anisotropy could be modeled and fitted instead. Since *R2a* and *R2i* (and *T2tot*) are fitted, they do not face the same issue of consistent underestimation.

OD of Safranin O-stained sections was found to increase with the cartilage OARSI grade. We consider this a surprising result since it contradicts both expectations and previously reported results. OD is generally thought to increase in proportion to PG content [21, 31]. However, safranin-O staining also binds to collagen crosslinks, which appear as the cartilage matures, which may cause inflation in the measured DD values without increase in PG content [40]. In addition, we find it surprising that OD correlated with *T1* and *R2i* in bulk, but not in any of the cartilage zones. Small variation in sample degeneration level and great spatial inhomogeneity in OD in the samples should be considered when assessing the reliability of this result. However, three slices were scanned and averaged to improve the reliability of OD quantification. Furthermore, different OA phenotypes can impose different changes in cartilage OD. This can be observed as a variation in data especially when the sample set of this study consists of several osteochondral plugs of the same person.

Unlike PLM or DD, qMRI can detect signal from unbound water in the tissue, which we speculate to explain its capability to differentiate the cartilage groups in the SZ. This is an aspect that should be studied in the future.

### Limitations

Re-orientation measurements are challenging to conduct, especially *in vivo*. In this study, extensive number of orientations were applied, however similar fairly reliable measurements can be done with substantially fewer angles [19]. Feasibility to conduct similar rotation studies with clinical scanners has also been demonstrated [41].

Although significant changes in qMRI parameters were observed in the superficial zone, it is also the zone most prone to measurement errors since it is the thinnest zone. Particularly in this study, possible sources of uncertainty include partial volume effects (including segmentation) and co-registration inaccuracies due to the multiple orientations scanned, necessitating co-registration of the imaging data prior to analyses. It is also important to note that the co-registration artifacts do not affect the *T1* and *T1ρ* measurements, which were acquired at a single orientation matching the typical *in vivo* scanning orientation for weight-bearing articular cartilage. The most significant sources of error for histological reference methods are mainly the slice thickness and position.

The age distribution of the cadavers in this study is skewed toward older individuals, which may limit the generalizability of these findings. In addition, similar results, possibly of greater significance, could be expected if the sample set included more severely degenerated samples. The samples were harvested from various load-bearing and non-load-bearing locations of femoral cartilage, introducing additional variation in the measured properties. Four of the individuals had more than one OARSI category samples demonstrating variation between samples even within the same individual which demonstrates the complexity of diagnosing OA.

## Conclusion

In this study, we demonstrated that isotropic and anisotropic components of *T2* are sensitive to degenerative changes in human articular cartilage. Unlike *T1* and *T1ρ* based parameters, *T2tot* and *R2a* correlated to phase shift between stress and strain during dynamic mechanical loading, which in turn showed to be sensitive to degenerative changes in cartilage. However, the results suggest that *T1* and *T1ρ* may be more suitable qMRI methods for characterizing tissue properties due their better sensitivity to degenerative changes and relative ease in scanning, as well as their reduced sensitivity to scanning orientation [27]. We conclude that while qMRI has great potential in the search for new diagnostic methods for early detection of OA, *T2* component mapping will likely have more applicability on the tissue research rather than as a diagnostic tool.

## References

[1] V. C. Mow, W. Y. Gu, and F. H. Chen, *Structure and function of articular cartilage and meniscus, Ch 5: Basic orthopaedic biomechanics and mechano-biology*, 3rd ed. Wolters Kluwer Health, 2005.

[2] Nissi, M. J., et al. T2 relaxation time mapping reveals age- and species-related diversity of collagen network architecture in articular cartilage. *Osteoarthritis Cartilage*. 14(12):1265–1271, 2006. 10.1016/j.joca.2006.06.002. 16843689 10.1016/j.joca.2006.06.002

[3] Courties, A., I. Kouki, N. Soliman, S. Mathieu, and J. Sellam. Osteoarthritis year in review 2024: Epidemiology and therapy. *Osteoarthritis Cartilage*. 32(11):1397–1404, 2024. 10.1016/j.joca.2024.07.014. 39103081 10.1016/j.joca.2024.07.014

[4] Buckwalter, J. A., and H. J. Mankin. Articular cartilage. Part II: Degeneration and osteoarthrosis, repair, regeneration, and transplantation. *J. Bone Jt. Surg.—Ser. A.* 1997. 10.2106/00004623-199704000-00022.

[5] Chu, C. R., A. A. Williams, C. H. Coyle, and M. E. Bowers. Early diagnosis to enable early treatment of pre-osteoarthritis. *Arthritis Res. Ther.* 2012. 10.1186/ar3845. 22682469 10.1186/ar3845 PMC3446496

[6] Regatte, R. R., S. V. S. Akella, A. Borthakur, J. B. Kneeland, and R. Reddy. Proteoglycan depletion-induced changes in transverse relaxation maps of cartilage: comparison of T2 and T1rho. *Acad. Radiol.* 9(12):1388–1394, 2002. 10.1016/s1076-6332(03)80666-9. 12553350 10.1016/s1076-6332(03)80666-9

[7] Fan, X., et al. Functional mass spectrometry imaging maps phospholipase-A2 enzyme activity during osteoarthritis progression. *Theranostics*. 13(13):4636–4649, 2023. 10.7150/thno.86623. 37649605 10.7150/thno.86623 PMC10465221

[8] Nieminen, M. T., et al. Quantitative MR microscopy of enzymatically degraded articular cartilage. *Magn. Reson. Med.* 43(5):676–681, 2000. 10.1002/(SICI)1522-2594(200005)43:5%3c676::AID-MRM9%3e3.0.CO;2-X. 10800032 10.1002/(sici)1522-2594(200005)43:5<676::aid-mrm9>3.0.co;2-x

[9] Knecht, S., B. Vanwanseele, and E. Stüssi. A review on the mechanical quality of articular cartilage—implications for the diagnosis of osteoarthritis. *Clin. Biomech. Bristol Avon*. 21(10):999–1012, 2006. 10.1016/j.clinbiomech.2006.07.001. 10.1016/j.clinbiomech.2006.07.001 16979270

[10] David-Vaudey, E., S. Ghosh, M. Ries, and S. Majumdar. T2 relaxation time measurements in osteoarthritis. *Magn. Reson. Imaging*. 22(5):673–682, 2004. 10.1016/J.MRI.2004.01.071. 15172061 10.1016/j.mri.2004.01.071

[11] Bear, D. M., A. Williams, C. T. Chu, C. H. Coyle, and C. R. Chu. Optical coherence tomography grading correlates with MRI T2 mapping and extracellular matrix content. *J. Orthop. Res.* 28(4):546–552, 2010. 10.1002/jor.20998. 19834953 10.1002/jor.20998 PMC5823006

[12] Rieppo, J., J. Hallikainen, J. S. Jurvelin, I. Kiviranta, H. J. Helminen, and M. M. Hyttinen. Practical considerations in the use of polarized light microscopy in the analysis of the collagen network in articular cartilage. *Microsc. Res. Tech.* 71(4):279–287, 2008. 10.1002/jemt.20551. 18072283 10.1002/jemt.20551

[13] Xia, Y. Relaxation anisotropy in cartilage by NMR microscopy (μMRI) at 14-μm resolution. *Magn. Reson. Med.* 39:941–949, 1998. 10.1002/mrm.1910390612. 9621918 10.1002/mrm.1910390612

[14] Kneeland, J. B. Articular cartilage and the magic angle effect. *Am. J. Roentgenol.* 177(3):671–672, 2001. 10.2214/ajr.177.3.1770671. 11517069 10.2214/ajr.177.3.1770671

[15] Erickson, S. J., R. W. Prost, and M. E. Timins. The ‘magic angle’ effect: background physics and clinical relevance. *Radiology*. 188(1):23–25, 1993. 10.1148/radiology.188.1.7685531. 7685531 10.1148/radiology.188.1.7685531

[16] Furman, G. B., V. M. Meerovich, and V. L. Sokolovsky. Correlation of transverse relaxation time with structure of biological tissue. *J. Magn. Reson.* 270:7–11, 2016. 10.1016/j.jmr.2016.06.018. 27380185 10.1016/j.jmr.2016.06.018

[17] Momot, K. I., J. M. Pope, and R. M. Wellard. Anisotropy of spin relaxation of water protons in cartilage and tendon. *NMR Biomed.* 23(3):313–324, 2010. 10.1002/nbm.1466. 20013798 10.1002/nbm.1466

[18] Zheng, S., Y. Xia, and F. Badar. Further studies on the anisotropic distribution of collagen in articular cartilage by μMRI. *Magn. Reson. Med.* 65:656–663, 2011. 10.1002/mrm.22648. 20939069 10.1002/mrm.22648 PMC3021642

[19] Leskinen, H. P. P., N. E. Hänninen, and M. J. Nissi. T _2_ orientation anisotropy mapping of articular cartilage using qMRI. *Phys. Med. Biol.*68(8):085004, 2023. 10.1088/1361-6560/acc169. 10.1088/1361-6560/acc169 36867883

[20] Mantripragada, V. P., W. Gao, N. S. Piuzzi, C. D. Hoemann, G. F. Muschler, and R. J. Midura. Comparative assessment of primary osteoarthritis progression using conventional histopathology, polarized light microscopy, and immunohistochemistry. *Cartilage*. 13(1 Suppl):1494S-1510S, 2021. 10.1177/1947603520938455. 32659115 10.1177/1947603520938455 PMC8808935

[21] Kiviranta, I., J. Jurvelin, M. Tammi, A. M. Säämänen, and H. J. Helminen. Microspectrophotometric quantitation of glycosaminoglycans in articular cartilage sections stained with Safranin O. *Histochemistry*. 82(3):249–255, 1985. 10.1007/BF00501401. 2581923 10.1007/BF00501401

[22] Ebrahimi, M., et al. Elastic, dynamic viscoelastic and model-derived fibril-reinforced poroelastic mechanical properties of normal and osteoarthritic human femoral condyle cartilage. *Ann. Biomed. Eng.* 49(9):2622–2634, 2021. 10.1007/s10439-021-02838-4. 34341898 10.1007/s10439-021-02838-4 PMC8455392

[23] Fugazzola, M., et al. Composition, architecture and biomechanical properties of articular cartilage in differently loaded areas of the equine stifle. *Equine Vet. J.* 2023. 10.1111/evj.13960. 37376723 10.1111/evj.13960

[24] Mohammadi, A., et al. Site- and zone-dependent changes in proteoglycan content and biomechanical properties of bluntly and sharply grooved equine articular cartilage. *Ann. Biomed. Eng.* 50(12):1787–1797, 2022. 10.1007/s10439-022-02991-4. 35754073 10.1007/s10439-022-02991-4 PMC9794534

[25] Linus, A., et al. Visible and near-infrared spectroscopy enables differentiation of normal and early osteoarthritic human knee joint articular cartilage. *Ann. Biomed. Eng.* 51(10):2245–2257, 2023. 10.1007/s10439-023-03261-7. 37332006 10.1007/s10439-023-03261-7 PMC10518273

[26] Klein, S., M. Staring, K. Murphy, M. A. Viergever, and J. P. W. Pluim. Elastix: A toolbox for intensity-based medical image registration. *IEEE Trans. Med. Imaging*. 29(1):196–205, 2010. 10.1109/TMI.2009.2035616. 19923044 10.1109/TMI.2009.2035616

[27] Hänninen, N., J. Rautiainen, L. Rieppo, S. Saarakkala, and M. J. Nissi. Orientation anisotropy of quantitative MRI relaxation parameters in ordered tissue. *Sci. Rep.* 7(7):1–11, 2017. 10.1038/s41598-017-10053-2. 28852032 10.1038/s41598-017-10053-2 PMC5574987

[28] Xia, Y., J. B. Moody, and H. Alhadlaq. Orientational dependence of T2 relaxation in articular cartilage: A microscopic MRI (μMRI) study. *Magn. Reson. Med.* 48(3):460–469, 2002. 10.1002/mrm.10216. 12210910 10.1002/mrm.10216

[29] Xia, Y., J. B. Moody, N. Burton-Wurster, and G. Lust. Quantitative in situ correlation between microscopic MRI and polarized light microscopy studies of articular cartilage. *Osteoarthritis Cartilage*. 9(5):393–406, 2001. 10.1053/joca.2000.0405. 11467887 10.1053/joca.2000.0405

[30] Witschey, W. R. T., et al. Artifacts in T1 rho-weighted imaging: compensation for B(1) and B(0) field imperfections. *J. Magn. Reson. San Diego Calif 1997*. 2007. 10.1016/j.jmr.2007.01.015. 10.1016/j.jmr.2007.01.015 PMC1995435 17291799

[31] Király, K., et al. Application of selected cationic dyes for the semiquantitative estimation of glycosaminoglycans in histological sections of articular cartilage by microspectrophotometry. *Histochem. J.* 28(8):577–590, 1996. 10.1007/BF02331378. 8894661 10.1007/BF02331378

[32] Rieppo, J., et al. Structure-function relationships in enzymatically modified articular cartilage. *Cells Tissues Organs*. 175(3):121–132, 2003. 10.1159/000074628. 14663155 10.1159/000074628

[33] Jäntti, J., et al. Cationic tantalum oxide nanoparticle contrast agent for micro computed tomography reveals articular cartilage proteoglycan distribution and collagen architecture alterations. *Osteoarthritis Cartilage*. 2023. 10.1016/j.joca.2023.11.020. 38061579 10.1016/j.joca.2023.11.020

[34] Pritzker, K. P. H., et al. Osteoarthritis cartilage histopathology: grading and staging. *Osteoarthritis Cartilage*. 14(1):13–29, 2006. 10.1016/j.joca.2005.07.014. 16242352 10.1016/j.joca.2005.07.014

[35] Rautiainen, J., et al. Multiparametric MRI assessment of human articular cartilage degeneration: Correlation with quantitative histology and mechanical properties. *Magn. Reson. Med.* 74(1):249–259, 2015. 10.1002/mrm.25401. 25104181 10.1002/mrm.25401 PMC4320684

[36] Kajabi, A. W., et al. Multiparametric MR imaging reveals early cartilage degeneration at 2 and 8 weeks after ACL transection in a rabbit model. *J. Orthop. Res. Off. Publ. Orthop. Res. Soc.* 38(9):1974–1986, 2020. 10.1002/jor.24644. 10.1002/jor.24644 32129515

[37] Truhn, D., et al. Differentiation of human cartilage degeneration by functional MRI mapping-an ex vivo study. *Eur. Radiol.* 29(12):6671–6681, 2019. 10.1007/s00330-019-06283-9. 31187218 10.1007/s00330-019-06283-9

[38] Wang, L., and R. R. Regatte. Quantitative mapping of human cartilage at 3.0T: parallel changes in T₂, T₁ρ, and Dgemric. *Acad. Radiol.* 2014. 10.1016/j.acra.2013.12.010. 24594416 10.1016/j.acra.2013.12.010 PMC3949430

[39] Kajabi, A. W., et al. Evaluation of articular cartilage with quantitative MRI in an equine model of post-traumatic osteoarthritis. *J. Orthop. Res.* 39(1):63–73, 2021. 10.1002/jor.24780. 32543748 10.1002/jor.24780 PMC7818146

[40] Rautiainen, J., et al. Effect of collagen cross-linking on quantitative MRI parameters of articular cartilage. *Osteoarthritis Cartilage*. 24(9):1656–1664, 2016. 10.1016/j.joca.2016.04.017. 27143363 10.1016/j.joca.2016.04.017

[41] Kantola, V., et al. Anisotropy of T2 and T1ρ relaxation time in articular cartilage at 3 T. *Magn. Reson. Med.* 92(3):1177–1188, 2024. 10.1002/mrm.30096. 38558167 10.1002/mrm.30096

